# Cerebrovascular manifestations of herpes simplex virus infection of the central nervous system: a systematic review

**DOI:** 10.1186/s12974-019-1409-4

**Published:** 2019-01-29

**Authors:** Larissa Hauer, Slaven Pikija, Eva C. Schulte, Laszlo K. Sztriha, Raffaele Nardone, Johann Sellner

**Affiliations:** 1 Department of Psychiatry, Psychotherapy and Psychosomatics, Christian Doppler Medical Center, Paracelsus Medical University, Salzburg, Austria; 2 Department of Neurology, Christian Doppler Medical Center, Paracelsus Medical University, Ignaz-Harrer-Str. 79, 5020 Salzburg, Austria; 3 Department of Neurology, Friedrich-Baur Institute, University Hospital of the Ludwig-Maximilians-Universität München, Munich, Germany; 4 Department of Neurology, King’s College Hospital, Denmark Hill, London, UK; 5 Division of Neurology, Franz Tappeiner Hospital, Merano, Italy; 6 Department of Neurology, Klinikum rechts der Isar, Technische Universität München, Munich, Germany

**Keywords:** Cerebrovascular complications, Herpes simplex virus encephalitis, Ischemic stroke intracerebral hemorrhage, Vasculopathy, Vasculitis, Mortality

## Abstract

**Background:**

Intracerebral hemorrhage and ischemic stroke are increasingly recognized complications of central nervous system (CNS) infection by herpes simplex virus (HSV).

**Aim of the study:**

To analyze clinical, imaging, and laboratory findings and outcomes of cerebrovascular manifestations of HSV infection.

**Methods:**

Systematic literature review from January 2000 to July 2018.

**Results:**

We identified 38 patients (median age 45 years, range 1–73) comprising 27 cases of intracerebral hemorrhage, 10 of ischemic stroke, and 1 with cerebral venous sinus thrombosis. Intracerebral hemorrhage was predominantly (89%) a complication of HSV encephalitis located in the temporal lobe. Hematoma was present on the first brain imaging in 32%, and hematoma evacuation was performed in 30% of these cases. Infarction was frequently multifocal, and at times preceded by hemorrhage (20%). Both a stroke-like presentation and presence of HSV encephalitis in a typical location were rare (25% and 10%, respectively). There was evidence of cerebral vasculitis in 63%, which was exclusively located in large-sized vessels. Overall mortality was 21% for hemorrhage and 0% for infarction. HSV-1 was a major cause of hemorrhagic complications, whereas HSV-2 was the most prevalent agent in the ischemic manifestations.

**Conclusion:**

We found a distinct pathogenesis, cause, and outcome for HSV-related cerebral hemorrhage and infarction. Vessel disruption within a temporal lobe lesion caused by HSV-1 is the presumed mechanism for hemorrhage, which may potentially have a fatal outcome. Brain ischemia is mostly related to multifocal cerebral large vessel vasculitis associated with HSV-2, where the outcome is more favorable.

## Introduction

Herpes simplex virus (HSV) is the cause of acute viral encephalitis in 50–70% of cases where a virus can be identified [1]. Both HSV-1 and HSV-2 can cause encephalitis (HSE), which is predominantly located in the temporal lobes and the limbic system. From a histopathological viewpoint, HSE is a necrotizing process with perivascular inflammation and edema. Clinical care guidelines since the 1980s have emphasized increased awareness and swift initiation of treatment with acyclovir in encephalitic syndromes, lowering the mortality to below 20% [2, 3]. HSE, however, continues to result in substantial overall morbidity and mortality [4, 5]. Half of the survivors experience moderate to severe disability after 1 year [6]. Their quality of life is significantly impacted by epilepsy, psychiatric disorders, cognitive problems, dementia, headache, and alcohol abuse [5, 7, 8].

Cerebrovascular disease occurs as a complication of a variety of central nervous system (CNS) infections [9]. Many patients with infectious cerebral vasculopathies require intensive care with a generally poor prognosis [10, 11]. A recent analysis of 4871 cases of HSE in the USA reported intracranial hemorrhage in 2.7% and ischemic stroke in 5.6% [12]. However, there has been no systematic study conducted to evaluate clinical presentations, causative agents, pathogenesis, and outcome of cerebrovascular complications in HSE. Most knowledge about HSV-related cerebrovascular disease is provided by case reports. Therefore, an appraisal of the current state of understanding in this field is much needed. We systematically studied HSV-related cerebral vasculopathies reported in the literature.

## Methods

We conducted a systematic review of medical literature to identify all published cases of cerebrovascular manifestations of HSV using MEDLINE/PubMed, Web of Science, and Google Scholar. The study period was January 2000 to July 2018. There were no language restrictions; non-English articles were included and translated using online resources such as Google Translate. Search terms used were “HSV,” “herpes,” “herpetic,” “meningoencephalitis,” or “encephalitis” and one of the following terms: “ischemia,” “infarction,” “stroke,” “hemorrhage,” “hematoma,” “vasculopathy,” or “vascular complication.” We reviewed titles, abstracts, and full articles. References in each identified article were reviewed to identify additional cases.

**Fig. 1:**
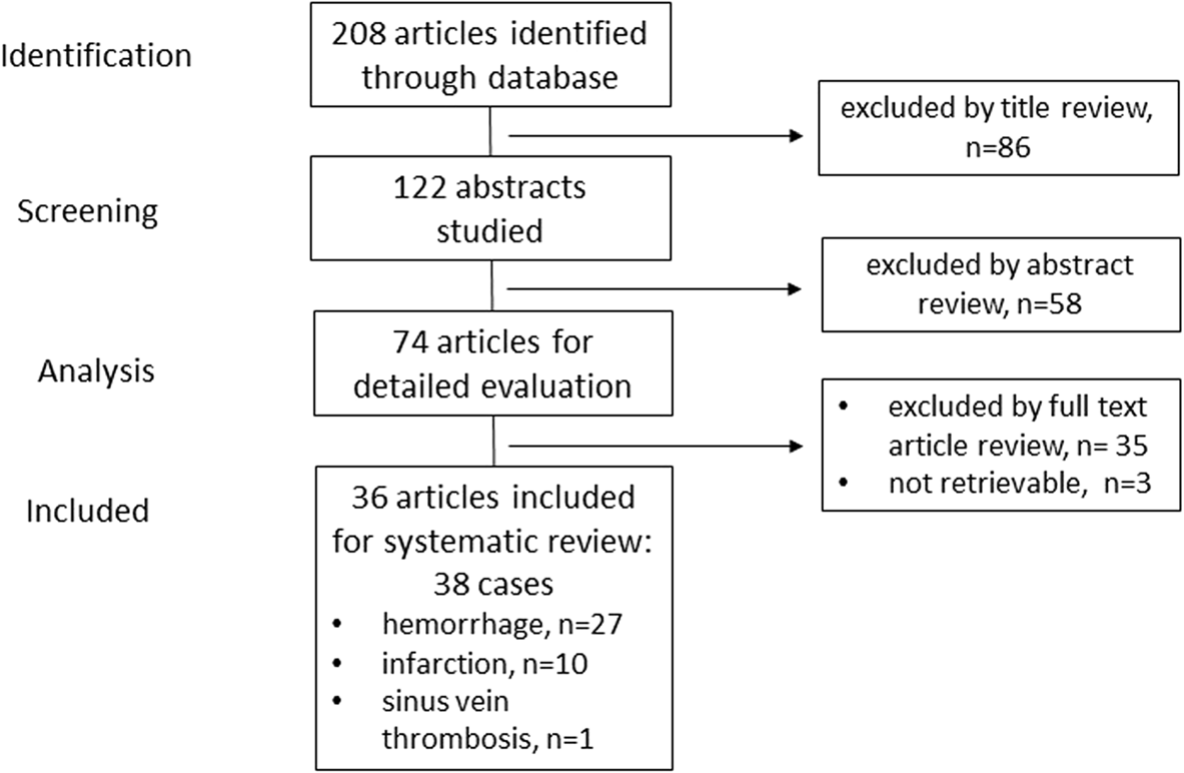
Flow chart of patient selection

The inclusion criteria were (1) radiological evidence of cerebrovascular manifestations of HSV (infarction, hemorrhage, or vasculopathy characterized by features of vasculitis, thrombosis, or aneurysm) by computed tomography (CT) or magnetic resonance imaging (MRI), (2) mandatory confirmation of HSV infection by analysis of cerebrospinal fluid according to the diagnostic criteria proposed in a recent consensus paper [13], and (3) exclusion of other causes for stroke. We limited our search to publications addressing HSE in children and adults; thus, neonatal cases were excluded. Details of the evaluation and selection process are shown in Fig. 1.

We extracted the following data: demographics, time from onset of symptoms to admission, neurological symptoms at presentation (classification: encephalitis, meningitis, stroke, other), and presence of imaging surrogates of cerebrovascular disease on admission and during the acute course. Stroke was defined according to the World Health Organization as “rapidly developing clinical signs of focal (or global) disturbance of cerebral function, with symptoms lasting 24 h or longer or leading to death, with no apparent cause other than of vascular origin.” Data on imaging included modality (CT or MRI), presence of encephalitis in a typical location (frontal or temporal lobe), characteristics of hemorrhage or ischemic lesion (unifocal or multifocal), distribution within vascular territories (anterior or posterior circulation or both), and features suggestive of vasculitis (small or large vessels or both). CSF data included cell count and the technique used to confirm CNS HSV infection (PCR, antibody, histology). Large vessel disease refers to involvement of the internal carotid artery (ICA), the vertebral artery (VA), the anterior cerebral artery (ACA) and its main branches, the middle cerebral artery (MCA) and its main branches, the posterior cerebral artery (PCA), and the basilar artery (BA) and its main branches [14]. Small vessels included the small penetrating arteries (e.g., the lenticulostriate arteries) that supply the deep structures of the brain. We also studied the use of steroids, as well as significant comorbidities. Outcome was classified according to the modified Rankin scale (mRS). Good outcome was defined as an mRS score of 0–2, and poor outcome as 3–5. Fatality (mRS 6) was categorized as an additional subgroup.

Statistical analysis was performed using the GraphPad Prism 7 software (La Jolla, CA).

## Results

### Systematic review

Details of the selection process are outlined in Fig. 1. We were unable to include three potentially relevant manuscripts as they were not accessible. These papers exclusively reported hemorrhagic manifestations of HSE [15–17]. We eventually analyzed a total of 36 manuscripts comprising 38 patients (Fig. 1). There were 27 cases of intracerebral hemorrhage [18–42], 10 with cerebral infarction [43–52], and 1 patient with venous sinus thrombosis [53].

### General and comparative analysis

The median age of the reported patients was 43 years (interquartile range (IQR) 27–65), and 19 (50%) were male. Six patients (15%) were younger than 18 years. There were no statistical differences of age and gender between patients with hemorrhagic and ischemic complications. Among cases where PCR distinguished between HSV-1 and HSV-2, HSV-1 was the predominant virus identified in cases with intracerebral hemorrhage (16/18, 89%). In contrast, cases with infarction were dominated by HSV-2 (5/7, 71%). There were patients with PCR-confirmed HSV CNS infection in whom the methodology did not distinguish between the two types (26% in the hemorrhage and 20% in the infarction group). Infectious comorbidities in patients with hemorrhage included HIV (*n* = 1) and hepatitis C (*n* = 1). There was a single case of systemic lupus erythematosus and immunosuppression in the infarction group. Demographics, clinical presentation, imaging, and outcome are presented separately for patients with hemorrhage and ischemia in Tables 1 and 2, respectively.

**Table 1:** Overview of demographics, clinical, and radiological findings in patients with hemorrhagic manifestations of HSV CNS infection

Demographics	
*n*	27
Median age (IQR), years	40 (26–54)
< 18 years	23% (4/27)
Male sex	55% (15/27)
Days from symptom onset to admission (median, IQR), *n* = 24	3.5 (2–7)
Clinical presentation	
Encephalitis	93% (25/27)
Stroke-like	4% (1/27)
Unspecific	4% (1/27)
Diagnostic testing	
HSV-1 (PCR of CSF)	59% (16/27)
HSV-2 (PCR of CSF)	7% (2/27)
HSV not distinguished (PCR of CSF)	26% (7/27)
HSV not distinguished (antibody of CSF)	4% (1/27)
PCR negative for HSV (CSF)	4% (1/27)
CSF findings	
Pleocytosis (> 4 cells/μl)	88% (21/24)
Median cell count^#^(cells/μl, IQR, *n* = 23)	88 (25–387)
Neuroimaging	
Hemorrhage on first imaging	32% (8/25)
Hemorrhage after admission	68% (17/25)
Days from admission to detection of hemorrhage (median, IQR)	10 (9–14)
Hemorrhage within HSE predilection sites	89% (24/27)
Bilateral temporal lobe HSE	33% (8/24)
Atypical localization of hemorrhage	7% (2/27)
No encephalitic lesion	4% (1/27)
Evidence for vasculitis	0% (0/9)
Intervention	
Hematoma evacuation	30% (8/27)
Outcome	
Good outcome (mRS 0–2)	38% (8/24)
Unfavorable outcome (mRS 3–5)	41% (11/24)
Fatality	21% (5/24)

^#^In patients with pleocytosis. *IQR* interquarile range

**Table 2:** Overview of demographics, clinical, and radiological findings in patients with ischemic manifestations of HSV CNS infection

Demographics	
*n*	10
Median age (IQR), years	47 (26–69)
< 18 years	20% (2/10)
Male gender	30% (3/10)
Days from symptom onset to admission	*n* = 3 on day 1, *n* = 1 on day 2
Clinical presentation	
Encephalitis	50% (5/10)
Stroke-like	30% (3/10)
Meningitis	20% (2/10)
Diagnostic testing	
HSV-1 (PCR of CSF)	20% (2/10)
HSV-2 (PCR of CSF)	50% (5/10)
HSV not distinguished (PCR of CSF)	20% (2/10)
PCR negative for HSV-1 (CSF) but antibody rise	10% (1/10)
CSF findings	
Pleocytosis (> 4 cells/μl)	100% (10/10)
Median cell count^#^ (cells/μl, IQR, *n* = 10)	42 (15–199)
Neuroimaging	
Ischemia on first brain imaging	50% (5/10)
Hemorrhage on first imaging, ischemia later	20% (2/10)
Presence of loco-typico HSV lesion	10% (1/10)
Evidence for vasculitis	63% (5/8)
Affected vessels	
Small-sized	0%
Large-sized	100% (5/5)
Affected areas of circulation	
Anterior	30% (3/10)
Posterior	20% (2/10)
Anterior and posterior	50% (5/10)
Distribution of lesions	
Single	10% (1/10)
Multiple	90% (9/10)
Intervention	
Steroid treatment	40% (4/10)
Outcome	
Good outcome (mRS 0–2)	56% (5/9)
Unfavorable outcome (mRS 3–5)	44% (4/9)
Fatality	0%

^#^In patients with pleocytosis. *IQR* interquartile range, *PCR* polymerase chain reaction, *CSF* cerebrospinal fluid, *HSV* herpes simplex virus

### Intracerebral hemorrhage

The clinical syndrome preceding admission was almost exclusively of encephalitis (93%). We found a median time lag of 3.5 days from symptom onset to hospital admission. The hematoma developed as a complication of HSV encephalitis in a typical location in most of the patients (89%). The parietal and occipital lobe, as well as deep brain structures, were the remaining locations of hematoma. The majority of bleedings were classified as parenchymal hemorrhage (*n* = 26), and only one case was petechial. Ventricular and/or subarachnoid blood was present in four patients. Many patients had cerebral edema, occasionally complicated by subsequent midline shift (*n* = 2), herniation (*n* = 4), or brainstem compression (*n* = 2). There was no evidence of a vasculitic pathology in the nine patients with vascular imaging and the three with histological examination of brain biopsy. No aneurysms were seen. Brain hemorrhage was detected on the first neuroimaging in eight patients (32%). More frequent was the development of hemorrhage after admission and the initiation of antiviral therapy (68%), with a time lag of a median of 10 days. Hematoma evacuation was performed in 30%. Outcome was unfavorable in 62%. The course of individual patients is presented in Table 3.

**Table 3:** Characteristics of patients with hemorrhagic manifestations of HSV CNS infection

No. (year), reference	Age, sex	HSV type, diagnostic test, CSF cells count	Initial clinical presentation, other findings	Localization of encephalitis	Localization of bleeding, other characteristics	Time from symptom onset to admission, time from admission to detection of hemorrhage	Presence of vasculitis (affected vessel), treatment	Outcome (mRS)
1 (2001), Plantinga E [18]	36, m	HSV-1, PCR, 33 cells/μl	Encephalitis (fever, consciousness, disorientation)	Left temporal, frontal, and insular areas	Left temporoparietal, hematoma, edema, midline shift	Day 6, day 10	n.e., dexamethasone	Mild aphasia and short-term memory disturbances (3)
2 (2001), Lee JW [19]	15, m	HSV*, PCR, 15 cells/μl	Encephalitis (fever, aphasia, confusion)	Both temporal and frontal lobes	Left temporal and frontal lobe, petechial hemorrhage	Day 4, on first imaging	n.e.	Weakness upper extremity (2)
3 (2001), Lee JW [19]	6, m	HSV*, PCR, 5 cells/μl	Encephalitis (fever, vomiting, altered consciousness)	Left medial temporal lobe	Left temporal and frontal lobe, hematoma	Day 17, on first imaging	n.e.	Severe bulbar dysfunction (4–5)
4 (2002), Erdem G [20]	1, f	HSV*, PCR, 33 cells/μl	Encephalitis (seizures, lethargy, fever)	Both temporal, frontal, and parietal lobes	Right temporal lobe, hematoma, edema	Day 6, on first imaging	No	Quadriparesis (4–5)
5 (2004), Biswas A [21]	38, m	HSV-1, PCR, 0 cells/μl	Encephalitis (headache, disturbed sleep)	Right inferior frontal and medial temporal region	Right frontal and temporal lobe, hematoma, edema	Not specified, not specified	No	Complete recovery (0)
6 (2005), Kabakus N [22]	3, m	HSV*, antibody, 450 cells/μl	Encephalitis (fever, headache hemiparesis)	Right temporal lobe	Left parietal lobe, hematoma, edema	Day 10, on first imaging	n.e.	Hemiparesis (3)
7 (2005), Jabbour PM [23]	27, m	HSV-1, PCR, 189 cells/μl	Encephalitis (fever, seizure)	Right mesial temporal lobe	Right temporal lobe, hematoma, uncal herniation, and ventricular blood	Day 2, day 9	n.e., craniotomy and evacuation of hematoma	No focal neurological signs (0)
8 (2006), Argyriou AA [24]	22, m	HSV-1, PCR, 425 cells/μl	Encephalitis (seizure, fever, altered consciousness)	Left termporal lobe	Left parietal lobe, hematoma	Day 3, day 11	n.e.	Complete recovery (0)
9 (2007), Shelley BP [25]	26, m	HSV*, PCR, 130 cells/μl	Encephalitis (fever, confusion, seizure)	Bilateral medial temporal lobe (left > right)	Left temporal lobe, hematoma	Day 1, day 18	no	Complete recovery (0)
10 (2008), Gkrania-Klotsas E [26]	46, m	HSV-1, PCR, 0 cells/μl	Encephalitis (headache, fever, depersonalization)	No lesion	Left parietal lobe, hematoma	Day 7, on first imaging	n.e.	Returned to premorbid mental condition (0–1)
11 (2009), Li JZ [27]	56, m	HSV-1, PCR, 30 cells/μl	encephalitis (fever, seizure), HIV positive	Left medial temporal lobe	Left temporal lobe and basal ganglia, hematoma, edema, herniation	Day 1, day 6 (no hemorrhage on CT on day 1)	n.e., craniotomy and evacuation of hematoma	Mild neuropsychological deficits (2)
12 (2010), Tonomura Y [28]	30, f	HSV-1, PCR, 321 cells/μl	Encephalitis (headache, fever, neuropsychological deficits, altered mental state)	Bilateral (left > right) medial temporal and frontal lobes	Left amygdaloid body, hematoma, subarachnoid, and ventricular blood	Day 2, day 5	n.e.	GCS 14, responds to simple orders (5)
13 (2011), Takeuchi S [29]	54, m	HSV-1, PCR, 86 cells/μl	Encephalitis (fever, walking difficulty, confusion, seizure)	Right temporal lobe	Temporal lobe, hematoma	Day 2, day 10	n.e.	Hemiparesis, memory disturbances (3)
14 (2013), Battaglia F [30]	38, f	HSV-1, PCR, pleocytosis	Encephalitis (headache, fever, hallucinations, speech disturbances)	Diffuse cerebral edema on CT	Left temporal lobe, hematoma, edema with brainstem compression	Day 3, day 9	n.e., craniotomy and evacuation of hematoma	Died 20 days from symptom onset (6)
15 (2013), Rodriguez-Sainz A [31]	45, f	HSV-1, PCR, 383 cells/μl	Encephalitis (headache, fever, incoherent speech), hepatitis C infection	Left medial temporal lobe	Left temporal lobe, hematoma, edema, brainstem compression	Day 2, day 9 (no lesion and no hemorrhage on CT on day 1)	n.e., craniotomy and evacuation of hematoma	Residual aphasia and right-sided hemiparesis (3–4)
16 (2013), Rodriguez-Sainz A [31]	53, f	HSV-1, PCR, 516 cells/μl	Encephalitis (fever, memory problems, headache)	Bilateral temporal lobes and insula	Left temporal lobe, hematoma, blood in subarachnoid space and midline shift	Day 6, day 8	n.e.	Mild neuropsychological deficits (3)
17 (2014), Yu W [32]	64, f	HSV*, PCR and brain biopsy, not reported	Encephalitis (headache, leg pain, seizure 5 days later)	Bilateral temporal and frontal lobe	Right temporal lobe, hematoma, raised intracranial pressure	No details, on first imaging	No, craniotomy and evacuation of hematoma	Died on hospital day 25 (6)
18 (2015), Zabroug S [33]	28, f	HSV-1, PCR, 2/μl	Encephalitis (anterograde amnesia, insomnia), 4 month postpartum	Right temporal lobe	Supra-tentorial blood	No details, no details	No	Not reported
19 (2015), Bhagchandania D [34]	23, m	HSV*, PCR, 20/μl	Encephalitis (fever, seizures, altered sensorium)	Bilateral temporal and parietal lobe	Left temporal lobe, hematoma	Day 4, day 15	n.e.	Behavioral abnormality (3–4)
20 (2016), Gaye NM [35]	53, f	HSV-2, PCR, 88 cells/μl	Encephalitis (recurrent seizures, fever on day 1)	Left mesial temporal lobe	Left temporal lobe, hematoma, ventricular blood	Day 2, day 18	n.e.	Persistent severe neuropsychological deficits (5)
21 (2017), Harada Y [36]	71, f	HSV-1, PCR, 170 cells/μl	Encephalitis (fever, headache, altered mental status)	Right anterior medial temporal lobe and insular cortex	Right temporal lobe and right basal frontal lobe, hematoma, intraventricular blood	Day 8, day 14	No	Near complete recovery (1)
22 (2016), Mahale RR [37]	71, m	HSV*, PCR, 5 cells/μl	Encephalitis (fever, headache, altered mental status)	No loco-typico lesions	Left parieto-occipital and right occipital region, hematoma	Day 5, on first imaging	n.e.	Mild improvement of cortical blindness (4)
23 (2016), Fisahn C [38]	69, f	HSV*, brain biopsy, not reported	Stroke-like (acute onset of headache and right hemiparesis)	No loco-typico lesions	Left parietal lobe, hematoma, subarachnoid hemorrhage	Day 1, on first imaging	n.e.	Died (6)
24 (2017), Mueller K [39]	40, f	HSV-2, PCR, 558 cells/μl	Unspecific (headache, fever, nausea, vomiting)	CT on first day normal	Right temporal lobe, hemorrhage, midline shift	Day 7, day 14	No, hemicraniectomy and temporal lobectomy	Survived, no further details reported
25 (2017), El Shimy G [40]	49, m	PCR negative, 45 cells/μl	Encephalitis (fever, headache, altered mental status)	Right temporal lobe and insula	Right medial temporal lobe, hematoma	Day 2, day 14	No	Returned to baseline neurological status (0)
26 (2013), Lo WB [41]	46, m	HSV-1, PCR, 390 cells/μl	Encephalitis (fever, headache, confusion)	Left temporal lobe	Left temporal lobe, hematoma, edema, uncal herniation	Day 7, day 10	Craniotomy on day 6, removal of anterior temporal lobe and evacuation of hematoma	Gradual improvement, no further details reported
27 (2018), Sivasankar C [42]	71, f	HSV-1, IHC, n.e.	Encephalitis (decreased responsiveness, hemiparesis, seizure)	Both temporal and parietal lobes	Right temporal lobe, hematoma, edema, uncal herniation	Day 1, day 8 (postoperative)	No, craniotomy for evacuation of hematoma on day 8	Died on day 17 after hospital admission (6)

*PCR methodology did not distinguish between HSV-1 and HSV-2. *m* male, *f* female, *+* yes, *−* no, *n.a.* not available, *CSF* cerebrospinal fluid, *MCA* middle cerebral artery, *MP* methylprednisone, *n.e.* not evaluated, *MRI* magnetic resonance imaging, *CT* computed tomography, *HIV* human immunodeficiency virus, *IHC* immunohistochemistry

### Ischemic stroke

The initial clinical presentations of patients in the infarction group included encephalitis (50%), meningitis (20%), and stroke (30%). No relevant comorbidities were reported; one patient was 2 months postpartum. Forty percent of the patients were admitted within 2 days from symptom onset, but this information was missing for three patients. Initial brain imaging was performed with CT in five, with MRI in four, and using both in one case. Brain infarction was detected on the first brain imaging in 50%, and an encephalitic lesion in a typical location was present in one patient (10%). Hemorrhage preceded infarction in two patients (20%), who were assigned to the group with ischemic manifestation due to the overlap of radiological features and pathogenesis. Multiple ischemic lesions were found in 90%, located most commonly in both anterior and posterior circulations. CSF pleocytosis was abundant in all patients. Vascular imaging was performed in eight patients, with evidence for cerebral vasculitis found in 63%. This exclusively involved the large arteries. Forty percent of patients with infarction were treated with steroids. Outcome was unfavorable in 40 %, but no one died. The course of individual patients is presented in Table 4.

**Table 4:** Characteristics of patients with ischemic manifestations of HSV CNS infection

No. (year), reference	Age, sex	HSV type, diagnostic test, CSF cell count	Clinical presentation, special features	Presence of loco-typico HSV-encephalitis	Time from symptom onset to admission, ischemia on first imaging	Presence of vasculitis (affected vessel), affected brain region	Steroids, dosage (duration)	Outcome (mRS)
1 (2004), Alexandri NM [43]	31, m	HSV-1, PCR, 46 cells/μl	Encephalitis (fever, hallucinations, headache)	Yes	Not reported, no (CT)	No, multifocal (anterior and posterior)	−	Hemianopsia (2)
2 (2009), Sas AM [44]	3, f	HSV-1, PCR, 38 cells/μl	Encephalitis (fever, impaired vision, nausea, vomiting)	No	Day 5, yes (CT and MRI)	No, multifocal (posterior)	−	Blindness (3)
3 (2012), Zepper P [45]	72, m	HSV-2, PCR, 588 cells/μl	Stroke-like (aphasia, hemiparesis)	No	Unclear, no (hemorrhage on CT)	Yes (various vessels), multifocal (anterior and posterior)	−	Moderate cognitive deficits and hemiparesis (3–4)
4 (2013), Guerrero WR [46]	36, f	HSV*, PCR, 13 cells/μl	Encephalitis (headache, diplopia with skew deviation), 2 months postpartum	No	Day 2, yes (MRI)	Yes (basilar artery), single lesion (posterior)	MP 3 days, P 7 days, dosages not reported	Internuclear ophthalmoplegia (3)
5 (2014), Terlizzi V [47]	10, f	HSV-1, IgG antibodies appeared in CSF, 20 cells/μl	Stroke-like (headache and hemiparesis)	No	Day 1, no (CT)	Yes (distal ICA and MCA), multifocal (anterior)	−	Complete recovery (0)
6 (2014), Snider SB [52]	57, f	HSV-2, PCR, 1260 cells/μl	Meningitis (headache, nausea, vomiting, photophobia)	No	Unclear, no (hemorrhage on CT)	Yes (various vessels), multifocal (anterior and posterior)	Yes, dosage not reported, 21 days	Minimal deficits (1–2), resolution of stenoses
7 (2016), Zis P [48]	45, m	HSV-2, PCR, 64 cells/μl	Encephalitis (fever, confusion)	No	Day 1, no (CT)	Not studied, multifocal (anterior)	−	Mild cognitive deficits (2)
8 (2016), Joshi P [49]	48, f	HSV-2, PCR, 16 cells/μl	Meningitis (fever, headache, neck stiffness), later encephalitis	No	Day 120, yes (MRI)	Yes (various vessels), multifocal (anterior and posterior)	MP and P dosage/duration not reported	Relapsing course, outcome not reported
9 (2017), Tsuboguchi S [50]	73, f	HSV*, PCR (6000 copies/ml), 8 cells/μl	Stroke-like (hemiparesis)	No	Day 1, yes (MRI)	Not studied, multifocal (anterior and posterior)	Yes, MP 500 mg, 3 days	Modified Rankin scale 5
10 (2017), Zhang F [51]	68, f	HSV-2, PCR, 649 cells/μl	Encephalitis (headache, numbness, hallucinations), systemic lupus, and immunosuppression	No	Day 21, yes (MRI)	No, multifocal (anterior)	−	Complete recovery (0)

*PCR methodology did not distinguish between HSV-1 and HSV-2. *m* male, *f* female, *+* yes, *−* no, *n.a.* not available, *CSF* cerebrospinal fluid, *MCA* middle cerebral artery, *MP* methylprednisone

### Venous sinus thrombosis

There was a single case of a 31-year-old man who had been suffering from fever, photophobia, and headache for 3 days. He was treated with acyclovir for suspected HSE (predominantly lymphocytic pleocytosis, HSV confirmed in CSF). Six days after admission, he developed bilateral upper limb weakness, ataxia, and bilateral headache. A brain MRI revealed features of encephalitis in the left temporal lobe and a superior sagittal sinus thrombosis. He was started on heparin and changed to warfarin for 1 year and was asymptomatic at follow-up.

## Discussion

On the basis of this systematic review of published cases, we propose that intracerebral hemorrhage and brain infarction are two pathogenetically distinct manifestations of CNS HSV infection. Intracerebral hemorrhage almost exclusively occurred within the temporal lobe, was related to HSV-1, and caused life-threatening complications. Intracerebral hemorrhage is most likely a complication secondary to disintegration of vessels in the context of a necrotic encephalitic process. In contrast, brain infarction is associated with HSV-2, pathomechanistically related to large-vessel vasculitis leading to multifocal ischemia, but with a less detrimental prognosis. Our study also revealed that the occurrence of cerebrovascular complications of CNS HSV infection is independent of age and gender and is not associated with comorbidities or immunosuppression.

All three alphaviruses (HSV-1, HSV-2, and varicella-zoster virus (VZV)) are dormant in trigeminal and upper cervical ganglia that innervate the cerebral arteries, dural sinuses, and other brain structures [54, 55]. Upon reactivation, these viruses can then travel transaxonally via an immunoprivileged route to infect the brain and arteries. HSE is a necrotizing inflammatory process typically affecting the cortex and underlying white matter of the temporal lobe [3]. The insula, cingulate gyrus, and posterior orbital frontal lobe are involved less frequently. Extra-temporal involvement is well described in HSE, to occur in more than half of the cases, sometimes even without any temporal abnormalities [56]. CNS pathology in HSE can be attributed to a combination of cytolytic viral replication and immune-mediated mechanisms leading to axonal and glial damage [57]. Histologic examination in acute HSE often shows cytotoxic and vasogenic edema as well as necrosis with petechial hemorrhages [58, 59]. Accordingly, limited hemorrhage is an integral part of the disease process, but in a subgroup of patients, a proper intracerebral hematoma develops. This complication is likely to result from weakening of the neurovascular unit caused by the necrotizing process via increased permeability due to cytokine, chemokine, and protease action [60–62]. Modi et al. reported a 2.7% rate of intracerebral hemorrhage in a large cohort of HSE patients, also associated with an increased mortality [12]. Here, we expand the understanding of this complication by reporting a frequent association of hemorrhage with severe edema, midline shift, herniation, and brain stem compression. Indeed, Jouan et al. reported high rates of ICU admission (32%) and mechanical ventilation (17%) in patients with HSE and a two-fold increase in mortality in those requiring ICU care [6]. However, the aforementioned study could not find any predictive features for brain herniation on the first neuroimaging. In our analysis, hemorrhage occurred in most patients after admission (68%) at a median of 10 days. This indicates that the processes leading to vessel disruption requires some time. Additional factors potentially increasing susceptibility for the development of a hematoma could include the vicinity of the encephalitic process to penetrating vessels, an impaired coagulation state, and the extent of the inflammatory response. A direct link between antiviral therapy and intracerebral hemorrhage cannot be ruled out as there are cases of acyclovir-induced thrombocytopenia [63, 64]. The presence of thrombocytopenia carried a six-fold increased risk of mortality in a cohort of encephalitis of any type [65]. Thus, both monitoring of coagulation and cautious use of anticoagulants may be advisable in severe encephalitis.

From the immunological viewpoint, an innate immune response driven by monocytes and natural killer cells dominates the early phase [66]. This response is followed by extravasation and parenchymal entry of T cells, particularly cytotoxic CD8+ T cells. On a molecular level, matrix metalloproteinase-9 (MMP-9), a major regulator of collagen type IV and the main component of the neurovascular matrix, has been recognized as a key factor in this process. In a mouse model of HSE, MMP-9 activity increased during the early phase with peak levels in fully developed HSE, resulting in compromise of the neurovascular unit [67]. This phenomenon is also known from hemorrhagically transformed ischemic stroke in humans, where an upregulation of MMP-9 has also been linked to disruption of the neurovascular unit. [68] A few studies proposed a beneficial role of adjuvant steroids in HSE, which may restrict detrimental immune responses by mechanisms such as inducing apoptosis of immune cells in peripheral blood and within the CNS, or neutralization of MMP-9 activity by raising tissue inhibitor of MMP-1 (TIMP-1) in vascular endothelial cells [69, 70].

Our study also revealed that HSE is an important differential diagnosis of lobar hemorrhage as 32% had a hemorrhage detected on initial neuroimaging. This emphasizes the need for sufficient medical history, as the patients almost exclusively presented with an encephalitic syndrome. Moreover, it should be noted that hemorrhage in the temporal lobe needs to be investigated with appropriate vascular imaging in order to rule out vascular malformations or other causes of atypical hemorrhage.

It has been recognized since the early 1970s that HSE can occasionally present with a cerebral infarction [71]. An epidemiological study of HSE from the USA reported ischemic complications occurring twice as frequently (5.6%) as hemorrhage. Both cases with hemorrhage as the presenting imaging feature and brain infarction later during the course were related to HSV-2 with evidence of vasculitis. HSV-1 was the causative agent in the only patient with classical temporal lobe encephalitis who developed infarction. There was only one case of brain stem infarction caused by HSV-2, which is another encephalitic predilection site of HSV-2. Imaging findings of vasculitis include stenosis, vessel wall thickening, and enhancement [72]. If knowledge from VZV vasculopathy can be translated, the lack of CSF pleocytosis or of angiographic abnormalities should not preclude the diagnosis of vasculopathy. Indeed, in VZV vasculopathy, up to 33% of cases may have normal CSF and 30% may have negative vascular imaging [73]. Thus, confirmation of vasculitis on neuroimaging should not be the decisive factor for considering adjunctive steroid treatment. Little is known about the exact mechanisms of action leading to HSV vasculitis. In case of VZV disease, cerebral vessels demonstrate fibrinoid necrosis, intimal proliferation, loss of elastic lamina, and a lymphocytic or monocytic inflammatory infiltrate with surrounding multinucleated giant cells [74]. Additional mechanism of action may include an immune-mediated process leading to vasospasm and thrombosis and hypercoagulable state in combination with endothelial dysfunction resulting from activation of inflammatory and pro-coagulant cascades [9].

We acknowledge several limitations to our study. A publishing bias for more severe and atypical cases must be anticipated. This might also explain higher rates of hemorrhage than infarction. In addition, imaging techniques and time points of investigations differed within our cohort. Furthermore, vasculitic change in small vessels may not be picked up by non-invasive angiography. Future studies should ideally employ MRI with standardized vascular imaging and a PCR methodology which differentiates between HSV-1 and HSV-2. There was only a single case of venous sinus thrombosis, for which significant conclusions cannot be drawn.

## Conclusion

We expand the understanding for HSV-related cerebrovascular disease and report distinct pathogenesis, cause, and outcome for cerebral hemorrhage and infarction. Further studies should focus on strategies to prevent intracerebral hemorrhage including avoidance of impaired coagulation and management of life-threatening consequences. In addition, HSV-related infarction is a rare but potentially treatable cause of stroke caused by large vessel vasculitis and usually does not clinically present as a stroke. Steroid treatment can be considered even in the absence of confirmation of vasculitis on neuroimaging.

## Abbrevations

CNS: Central nervous system
CT: Computer tomography
HSE: Herpes simplex virus encephalitis
HSV: Herpes simplex virus
ICU: Intensive care unit
MMP: Matrix metalloproteinase
MRI: Magnetic resonance imaging
mRS: Modified Rankin scale
PCR: Polymerase chain reaction
TIMP: Tissue-inhibitor of MMP
VZV: Varicella-zoster virus
